# Mobilisation of emergency services for chemical incidents in Sweden - a multi-agency focus group study

**DOI:** 10.1186/s13049-021-00910-5

**Published:** 2021-07-21

**Authors:** Anton Westman, Britt-Inger Saveman, Ulf Björnstig, Johan Hylander, Lina Gyllencreutz

**Affiliations:** 1 Department of Surgical and Perioperative Sciences, Surgery, Centre for Disaster Medicine, Umeå University, 901 87 Umeå, Sweden; 2 Department of Anesthesia and Intensive Care Medicine, Karolinska University Hospital, Huddinge, Sweden; 3 Department of Nursing, Umeå University, Umeå, Sweden

**Keywords:** Accident and emergency medicine, Public health, Emergency response and management, Emergency planning, Decision framework, Communication, Chemical incidents, Disasters

## Abstract

**Background:**

In chemical incidents, infrequent but potentially disastrous, the World Health Organization calls for inter-organizational coordination of actors involved. Multi-organizational studies of chemical response capacities are scarce. We aimed to describe chemical incident experiences and perceptions of Swedish fire and rescue services, emergency medical services, police services, and emergency dispatch services personnel.

**Methods:**

Eight emergency service organizations in two distinct and dissimilar regions in Sweden participated in one organization-specific focus group interview each. The total number of respondents was 25 (7 females and 18 males). A qualitative inductive content analysis was performed.

**Results:**

Three types of information processing were derived as emerging during acute-phase chemical incident mobilization: Unspecified (a caller communicating with an emergency medical dispatcher), specified (each emergency service obtaining organization-specific expert information), and aligned (continually updated information from the scene condensed and disseminated back to all parties at the scene). Improvable shortcomings were identified, e.g. randomness (unspecified information processing), inter-organizational reticence (specified information processing), and downprioritizing central information transmission while saving lives (aligned information processing).

**Conclusions:**

The flow of information may be improved by automation, public education, revised dispatcher education, and use of technical resources in the field. Future studies should independently assess these mechanism’s degree of impact on mobilisation of emergency services in chemical incidents.

**Supplementary Information:**

The online version contains supplementary material available at 10.1186/s13049-021-00910-5.

## Introduction

### Background

Chemical exposures are distinguished by equivocal characteristics. Toxic emissions may cause immediate or delayed symtoms, may be colorless, odorless and tasteless, may contaminate rescuers and environment, and may escalate to disaster if not contained. In 2009, the World Health Organization (WHO) stated that in the event of any chemical incident, a timely and robust mechanism is needed to mobilize responders [1]. The WHO noted that, since chemical incidents are both complex and acute, an optimal response can only be achieved through coordination of actors involved, stressing the importance of interoperability of communication equipment, procedures, and systems. Toxic emissions are relatively uncommon in Sweden, but serious incidents have occurred [2, 3]. Systematic research describing in detail the scale of the problem is lacking. By comparison, Sweden’s neighbour country Poland suffered 2930 chemical incidents during the time period 1999–2009, with more than 200 different substances released and 18 fatalities reported [4]. Directly translating the Polish numbers adjusted for population size suggests circa 70 chemical incidents per year in Sweden. This figure could be higher, since Sweden despite its smaller population has a higher number of establishments falling under the Seveso-II-Directive [5]. The implementation of new technologies and materials in buildings and vehicles, e.g. polyurethane and electric car traction batteries, may add new or increased toxicological risks in fires. Road tunnels and underground transportation hubs may contribute to toxicological exposure levels in vehicle fires [6], and in structure fires; discussions about the Grenfell Tower disaster in London 2017 have involved the choice of materials in relation to the cyanide poisoning of its residents [7]. There is also an awareness of the potential use of chemical agents in acts of terrorism [8]. Response capacity to outlier events has been suggested to rest on the back of strong day-to-day systems [9]. Thus, it would seem of interest to examine mechanisms to mobilize responders in chemical incidents.

Sweden has no uniform management structure for its emergency services. The emergency medical communication centers have a nationwide coverage of emergency calls and dispatch, but at a secondary level the rescue service and police also have their own communication hubs. The rescue service has a special focus and responsibility in chemical incidents, but all emergency organizations are responsible for their own contribution and own safety. The rescue service is regarded as having a leadership role, though not by legal definition. The emergency organizations are responsible for the plans that the emergency medical communication centers have to follow, and are also responsible for their own local or regional plans. In fact, the emergency organizations operate under different legislation and regulations [10]. The emergency organizations are expected to cooperate at the incident scene, but while full-scale exercises for other rare but potentially disastrous events have been conducted and evaluated, e.g. mining incidents [11], common training for major chemical incidents is to our knowledge very rare in Sweden. Previously, the general performance of Swedish emergency medical dispatch protocols have been evaluated from various perspectives [12, 13]. In the neighbouring country of Finland, the preparedness of the emergency medical services for chemical emergencies has been surveyed, finding unsufficient antidote and decontamination capacity [14]. A similar survey in the Netherlands found serious lack of hospital preparedness for chemical, biological, or radionuclear (CBRN) incidents [15]. Also in the Netherlands, a study of general coordination capacities of emergency services found patterns of organizational fragmentation emerging when challenged with disaster training [16].

### Importance

Literature on integrated inter-organizational response capacities to chemical incidents is largely lacking.

### Aim

The aim of this study was to describe experiences and perceptions of emergency services and emergency service dispatch personnel regarding chemical incident mobilisation.

## Methods

### Study design, setting, and selection of participants

This study has an inductive approach with interpretations of interviews, following the consolidated criteria for reporting qualitative studies (COREQ) [17]. Eight Swedish emergency service organizations, including fire and rescue services (RS), emergency medical services (EMS), police services (PS), as well as emergency dispatch services (EDS), were conveniently invited via e-mail to participate in one organization-specific focus group interview each. Each focus group interview was intended to consist of about five respondents and lasting 1-1.5 h. As the Swedish emergency services have regional diversity and a degree of national heterogeneity regarding terminology and systems of hierarchical relationships, we decided to do interviews by service instead of mixing them. The invited organizations were located in two regions in Sweden, one in the south and the other in the north. Both catchment areas contain heavy chemical industries, busy industrial ports, as well as roads and railways carrying hazardous materials. One of the study areas was characterized by chemical industries and the other by mining and forestry industries. This study was approved by the Swedish Ethical Review Authority (dnr 2019–02043) and was conducted in accordance with the Helsinki Declaration [18].

### Data collection

Interviews were conducted June through September, 2019. All interviews were conducted by the principal author. A semistructured interview guide was developed through iterative discussions among authors (Additional file 1). All interviews were held at the respective workplace during working hours in a separate room without anyone present besides the respondents and researchers. The audio-recorded interviews lasted between 44 and 90 min and were transcribed verbatim.

### Characteristics of study subjects and derived categories

All eight invited organizations chose to participate, including a specialist EMS for chemical incidents, the Chemical Ambulance (CA). The total number of respondents was 25 (7 females; 18 males). No respondent declined participation or dropped out. In two cases, only one respondent could participate - they both gave rich data to the study. Respondent experience levels are presented in Table 1.

**Table 1:** Characteristics of study subjects. Experience level (mean years of experience and standard deviation, SD) of interview respondents per geographical catchment area and emergency service organization

	Number of **respondents**	Years of experience **Mean (SD)**
**Catchment area A**		
Emergency dispatch services (EDS)	3	5 (6)
Fire and rescue services (RS)	4	13 (7)
Police services (PS)	3	11(1)
Emergency medical services (EMS)	1	16 (N/A)
**Catchment area B**		
Emergency dispatch services (EDS)	6	10 (9)
Fire and rescue services (RS)	4	10 (5)
Police services (PS)	1	14 (N/A)
Emergency medical services (EMS)	3	15 (11)
**TOTAL**	**25**	**11 (7)**

### Analysis

The text was analyzed using qualitative content analysis [19, 20], including iterative steps to enhance trustworthiness of the results. This method analyses both manifest (explicit) and latent (implicit) content, by grouping meaning units into subcategories, categories, and sometimes themes, with quotations used to describe internal consistency. In this study, emphasis was placed on the manifest content, i.e., what the respondents actually said. To mitigate analysis biases, initial coding was performed independently by the authors as follows: First, the authors read the entire material thoroughly several times to grasp the content. The principal author analyzed two interviews marking meaning units and codes relating to the aim of the study. The other authors independently made the same procedure with the rest of the interviews. Thereafter, categorizations were performed from all the codes leading to consensus about emergent sub-categories from which categories were derived. The respondents did not provide feedback on the findings.

## Results

Derived categories are presented in Table 2.

**Table 2:** Derived categories and underlying subcategories

Subcategories:	Main categories:
The incoming call	Unspecified information processing
Receiving, understanding and indexing	
Listening in and disseminating information	
Obtaining organization-specific information	Specified information processing
Risk assessment	
Building puzzles centrally	Aligned information processing
Continously updated information from the scene	

### Main category: Unspecified information processing

The derived category *Unspecified information processing* concerns how a caller communicates with the emergency medical dispatcher (EMD). It further concerns how the emergency medical communication center (EMCC) receives the incoming call, understands and indexes the information, as well as how experts listening in (added by EMD request to the call) are coordinated, and how the information is disseminated.

#### Subcategory: The incoming call

EMDs expect the incoming call to provide information about how the caller has perceived the situation. The information can be quite clear (e.g. a gas leak), but there were descriptions of misunderstandings. The caller may only have a fragmented understanding of the situation, while at the same time, there may be several callers who can provide complementary information. Some callers were perceived to show adaptive capacity to take action at the scene.

*The alarm was grass fire. True, when they arrived it sure was a grass fire, 20 × 30 m [next to] the railway… and also nine carriages laying there leaking.*

- EDS respondent, focus group #1.

#### Subcategory: Receiving, understanding and indexing

The initial information from an incoming call was said to only provide, at best, a partial description of the situation. The indexing process, by contrast, must rapidly identify and attach to the conversation personnel with the correct type of competence. The indexing process emphasizes automation. EMDs ask questions from the emergency dispatch index (EDI) interview guide, a criteria-based dispatch protocol structured in sub-indices, e.g. the medical index. In situations having a clear EDI, a swift response can be expected. Experiences from trucking incidents with a non-Swedish-speaking crew included insufficient cargo information. An EMD seeks to assess risk, and typically gives basic safety advice to callers. An open mind was perceived as needed to characterize an incident. One important variable mentioned was geographical position, perceived to be facilitated by local knowledge. The EMD must be able to adapt socio-linguistically to the caller, including dialectal nuances. Interviewing was considered an art learned through experience. In the event of multiple, simultaneous, incidents, it was perceived as difficult to know if they were related, since an EMD typically handles only one event at a time.

*Oops, I have drilled a hole in the ground, a hole in a pipeline … What kind? Well, it’s a gas pipeline … What kind of gas? Do you see… smell… describe!*

- EDS respondent, focus group #5.

*The driver may not know where he is. He may know that he has passed [city X] and has not yet come to [city Y].*

- RS respondent, focus group #2.

#### Subcategory: Listening in and disseminating information

The respondents described a rapid assembly and coordination of competence by listening in from senior personnel from both the RS and EMS. All parties become participants of the conversation. Listening in was considered beneficial to create inter-organizational consensus about the situation. In the view of the respondents, the information disseminated should contain facts about possible hazardous materials involved, life-saving needs, and an estimate of urgency.

A general experience of being called out to chemical incidents was initial uncertainty about hazardous materials. The PS were perceived by the other organizations to have access to restricted information (e.g. ongoing lethal violence). The Swedish national public warning system (Viktigt meddelande till allmänheten, VMA; Important public announcement, IPA) was perceived as functional and appropriately used.

The CA was if possible called out to the scene, but was also consulted for listening in to give telemedical advice. It was perceived that the CA was not contacted by default in all chemical incidents, and that the primary contact to the CA may be through another party than the EMCC, such as the RS calling directly from a scene. Lack of medical knowledge was considered problematic for an EMD, but help could be obtained from available nursing competence. An EMD cannot deploy too much resources to an incident while, by contrast, the emergency dispatch liaison officer (EDLO) may activate all available RS resources without regard to cost. By further contrast, the EDLOs were perceived as having a sufficient amount of indices and the mandate to deviate from these, and make decisions other than those predetermined.

*I have been to incidents where the police only afterwards told us it was a threatening situation.*

- RS respondent, focus group #6.

*A good alarm is when [the EMD] ‘catches’ the caller so that [we are told] what is written on the orange sign… Then we can search for information directly.*

- EMS respondent, focus group #4.

### Main category: Specified information processing

The derived category *Specified information processing* concerns the need for the emergency services, after receiving initial information from the EMCC, to collect additional information relevant for their own organization.

#### Subcategory: Obtaining organization-specific information

Additional information sought included hazardous materials involved and wind direction. A prime source of additional information mentioned was a national database and decision support service provided by the Swedish Civil Contingencies Agency - the respondents did not discuss this service in terms of any technical problems. The Swedish Poisons Information Centre also provided information, as well as general Internet search engines such as Google. Additional information was needed for EMS to be optimally distributed throughout the catchment area - in contrast to the RS’ approach to scale up initially and then withdraw excess resources. The expertise of the CA was perceived as contributing to optimal resource allocation, including being able to withdraw excess resources from a scene. It was perceived that the PS can share certain information with the CA that it cannot with ”ordinary” EMS personnel. The respondents perceived that specific information was needed to know which equipment was to be taken out to the scene. This was seen as problematic for the PS who does not always ride with chemical protection clothing in a typical police car, yet may be the first to arrive at the scene. Without organization-specific additional information, the respondents described a risk of moving up too close to an event. Emergency medical respondents described how they also can arrive at a scene before receiving additional information. It was described that if you are in a vehicle already moving, there was less time to gather information than if you are at the station. The national collaborative radio channel system was perceived as helpful in providing additional information.

*You often get information from the rescue service, they are key players.*

- PS respondent, focus group #3.

*The police, we are alerted late. We have calculated that on average the difference between when the rescue service gets the alarm and until we have a patrol that gets the alarm is 12 min.*

- PS respondent, focus group #7.

#### Subcategory: Risk assessment

Risk assessment information was perceived as obtained from the EMCC, from the other organizations, from people at the scene, by observing (e.g. with binoculars) from a distance, and by using drones. A chemical incident was considered a prime responsibility for the RS, though they were not always first to the scene. The PS and EMS trusted RS risk assessments. Risk assessments for urban vs. rural areas differ. Evacuation need was described as a function of risk assessment. The respondents expressed a view that personnel should not be endangered to save lives, yet do take excess risks sometimes. As part of the risk assessment, the level of personal protection needed and time limits to exposure are determined. It was perceived that actions taken by the PS must be given precedence.

*Should we arrive first knowing that lives are on the line, then we might skip the chemical suit since we can almost not move in it, and pull [the victims] out from the danger zone.*

- EMS respondent, focus group #4.

*We have seen the police go down into fires with filter masks and say that it takes everything. Yes, but it does not add oxygen.*

- RS respondent, focus group #6.

### Main category: Aligned information processing

The derived category *Aligned information processing* concerns the need for the EMCC to receive continually updated reports from the scene, in order to be able to disseminate a more complete overall situation description to all parties.

#### Subcategory: Building puzzles centrally

Throughout operations during a chemical incident, the EMCC was perceived as having the task of centrally collecting new information and thereafter fitting this information into the situation description, and continuously disseminate the updated description to all parties. This communication was described as sometimes unsatisfactory.

… *[the organizations] cannot have a similar perception of the situation [since] we work under completely different conditions.*

- PS respondent, focus group #7.

#### Subcategory: Continuously updated information from the scene

In contrast to the central need for information, it was perceived as difficult to continuously send updated information from an ongoing operation. Respondents thought that obtaining and transmitting such information was not possible while saving lives. Inter-organizational communication at the scene was perceived as, at times, unsatisfactory. Respondents described how easy it was to forget the other organizations while focusing on urgent own work. It was perceived that information from the PS needs to be actively requested. One perceived value of continuously updated information is that it may disseminate awareness about multiple, simultaneous, incidents.

*The police use their own [radio] channel and they do not always come over into ours. The ambulance has its own channel and rarely changes to a collaborative channel.*

- RS respondent, focus group #6.

*As soon as we get the [first unit] windshield report, then it supplements the information we received from the beginning. I do not feel we get as much response from the police, [it is] almost always just the ambulance and the rescue service.*

- EDS respondent, focus group #5.

## Discussion

### Main findings

While our respondents did not describe technical problems with communication systems, they perceived difficulties in centrally collecting continuous reports from the scene, and to disseminating that updated information to all engaged parties. In terms of the WHO recommendations, it appears that the *interoperability of communication equipment and systems* was considered unproblematic, but that *coordination of actors involved* has improvable variables. This is in line with a theoretical suggestion that disaster response interoperability degrades as number of actors increase [21].

### Unspecified information processing

The EMD receiving the initial incoming call has a formidable task. Information retrieval obstacles revealed include a fragmented understanding of the situation by the caller; caller-EMD language problems (including dialects); and the incomplete predictive capacity of the EDI interview guide. Types of chemical incidents having comparatively well-defined interview guides, e.g. gas leaks, are described as less challenging. This indicates a problem when the crucial first communication falls outside predicted scenarios. The initial incoming call can be received by any EMD, stochastically, regardless of experience. In Sweden, EMD education is 10–11 weeks [22]. In an evolving chemical disaster, the initial information retrieval is dependent on two human variables (the caller and the EMD) who both have properties of randomness and less-than-expert level of chemical competence. A countermeasure could be increased automation, e.g. revising current interview guides regarding chemical incidents or adding an automaton third party to the caller-EMD conversation in the form of artificial intelligence (AI). Limitations to AI usefulness should, however, be considered [23]. Recommendations from a recent report of Swedish EDS include standardizing terminology, developing technical solutions for information sharing and geodata systems, and organizational reform [24].

### Specified information processing

After the initial information has been passed on from the EMCC to the respective emergency services, a problem emergent is no longer randomness, but information access and literacy. All engaged parties need to obtain as much organization-specific information of as high quality and relevance as possible, as fast as possible. It has been noted that toxicologists must be ready to gain and interpret analytical data in the response phase, to support both medical care and repeated risk assessment [25]. It appears from our interviews that the most commonly used source of information at this stage is a database and decision support service provided by the Swedish Civil Contingencies Agency [26, 27]. However, the respondents also discussed other means of obtaining information, including Google - to date subject to third-party influence [28]. The view of respondents from other organizations that the PS not always release all information may be worth enquiry. The positive perception of the consultant role of the CA, i.e. incidents in which its personnel partakes only telemedically, suggests a broader implication of such specialist competence, possibly nationwide.

Risk assessment in chemical incidents is perceived as dependent on inter-organizational communication. Respondents considered the RS having prime responsibilities for risk assessment, but the service first arriving may be the EMS or PS. Conflicting statements emerged about when in the sequence of arrivals the PS typically enter the scene: On the one hand, the PS considers it probable to arrive first at an urban scene since they are patrolling in their vehicles and may be only a few minutes drive away. On the other hand, a deployment delay of up to twelve minutes between RS and PS is mentioned. Apart from a need to examine police deployment routines, this variability also indicates initial excess risk exposure for the PS. This is consistent with a study of first responders injured in acute chemical incidents in the USA 2002–2012, finding that police officers had rarely used personal protective equipment [29]. Firefighters were, however, most frequently injured. Our respondents described risk assessment information from various sources as being collected and processed by the EMCC (having engaged expertise centrally) and then disseminated, with the RS as a main addressee. In triage of chemical incident victims, the need to assess the risk of secondary contamination to emergency services must also be considered [30]. Our results show a high level of trust in the RS capacity to make risk assessments. However, it must be noted that this capacity is partially dependent on the capacity of the EMDs to continually receive updated information from the scene - which is described by all organizations as difficult.

### Aligned information processing

While there were no descriptions of technical problems with communication systems, EMDs described difficulties in receiving continuous reports from the scene, and in disseminating updated situation descriptions. This is not a unilateral view of EMD respondents - respondents working in the field confirm how difficult they find it to prioritize information transmission while saving lives. Previous literature notes how the needs of the injured take precedence over professional cross-border cooperation [31].

## Conclusions

It may be inferred from our results that during a chemical incident, the initial flow of information can be improved by automation, public education, revised dispatcher education, and use of technical resources in the field. An increased degree of EMCC automation may involve the development of purposeful artificial intelligence, e.g. trigger word detection and a predictive capacity for evolving chemical scenarios. Public education interventions may target select populations, e.g. professions. Possible interventions in dispatcher education and certification should be explored. All of these suggested variables, inferred from the present study, should be studied independently to assess possible impact on mobilisation of emergency services in chemical incidents.

## Limitations

Our own experiences of prehospital emergency healthcare entailed risk that we imposed our own views during interviews or were biased when coding. We sought to self-monitor and hold preunderstandings within brackets. Interviewing the Swedish emergency services separately was considered the best way to catch their own view of their job towards other services in a chemical incident. It was also a way to focus on their experiences instead of e.g. discussions of their various terminology. Making the analysis without consideration of the involved organizations would probably have resulted in some different results.

Even if there was a small number of participants, they gave rich descriptions of incidents they had experienced. The analysis and the findings received gave a good transferability to other contexts with similar professionals.

## Supplementary Information

as **Additional file 1:** Interview guide

## Data Availability

The datasets generated and analysed during the current study are not publicly available due to respondent integrity concerns.
