# Supplementary material for: Mobilisation of emergency services for chemical incidents in Sweden - a multi-agency focus group study
Source: Scand J Trauma Resusc Emerg Med. 2021 Jul 21;29:99. doi: 10.1186/s13049-021-00910-5 (PMC8293566; doi:10.1186/s13049-021-00910-5)
Supplement: Supplementary file 1 — Additional file 1: Interview guide [file 13049_2021_910_MOESM1_ESM.pdf]

Interview guide Mobilisation of emergency services for chemical incidents in Sweden - a multi-agency focus group study

Description of interview guide:

This interview guide was developed through iterative discussions among the authors and underwent ethical vetting by the Swedish Ethical Review Authority (dnr 2019-02043). The translation to English was made by the principal author and confirmed by all authors. The original Swedish interview guide is available on request.

English translation of interview guide:

* Oral verification of the content of the participant information. Introductory information and questions about professional experience and vocational training.

* In general - what are your routines for suspected "chemical incidents"?

* **Have you worked with alarms in the event of a suspected "chemical incident"? Examples, industrial fire, car fire?** (Comment: This is an important question that is expected to unlock discussions and / or a story about a specific event - develops into a narrative question.)

* How did it go with the alarm? What was said? Did you perceive special keywords / trigger words from callers? What is important in the communication between caller and emergency medical dispatcher?

* How is the emergency dispatch index interview guide structured for this type of event?

* Can you give examples of a good alarm? What factors contributed to its success?

* Can you give examples of when things did not go so well? What factors contributed to the failure?

* How does the collaboration between emergency medical communication center and blue-light organizations[1] take place? What is important in communication? All information to everyone? Connected in the event of a major event?

* Do you have any part in creating an initial perception of how dangerous the situation is? Has it been possible to create a common initial perception of the event for the emergency medical communication center and the blue-light organizations?

1In Sweden, the emergency service organizations are collectively referred to as the "blue-light organizations".

* How is your knowledge of hazardous substances and chemical risks in your catchment area?

* Have you been able to get information about special considerations and risks in the event of an incident?

* How do, in collaboration with the emergency medical communication center, blue-light units prepare during the alarm phase?

* Is there any technical support / equipment / system that is of particular value in this context? Is there a technical system that is missing and should be implemented / created?

* Could you in the group please discuss what, in your opinion, is important in your work with events like this?

* Could you in the group please discuss what, in your opinion, can be done to improve baseline conditions for your work?
